# The feasibility of antimicrobial lead time as process and quality indicator for hospitals

**DOI:** 10.1007/s10096-025-05085-w

**Published:** 2025-03-07

**Authors:** R. I. Helou, H. van der Sijs, D. Rizopoulos, M. Vogel, N. J. Verkaik, A. Verbon

**Affiliations:** 1 Department of Medical Microbiology and Infectious Diseases, Erasmus MC University Medical Center, Rotterdam, The Netherlands; 2 Department of Hospital Pharmacy, Erasmus MC University Medical Center, Rotterdam, The Netherlands; 3 Department of Biostatistics, Erasmus MC University Medical Center, Rotterdam, The Netherlands

**Keywords:** Antimicrobial lead time, Anti-infective agents, Drug prescriptions, Electronic prescribing, Quality indicator, Process indicator

## Abstract

**Purpose:**

Antimicrobial lead time (ALT) is the time from antimicrobial order to administration, an understudied parameter. This study aims to determine feasibility of retrieving ALT, differences in ALT for different infectious diseases and the association of ALT with length of stay (LoS) in order to establish the value of this parameter as potential new process or quality indicator (QI).

**Methods:**

In a retrospective study in a tertiary care hospital in the Netherlands, adult hospitalized patients treated for an infection were included over a 20-month period. ALT was calculated with data from the electronic health record system with computerized provider order entry.

**Results:**

Thousand patients (56.1% men, median age 61 years) were included. The median ALT was 1.05 h and significantly shorter in septic patients (*n* = 65) than in patients with other infections (*n* = 935; 0.27 h, interquartile range (IQR) 0.07–0.67 vs. 1.18 h, IQR 0.37–3.15; *p* < 0.001). If blood cultures were obtained median ALT was shorter (0.85 h vs. 1.77 h; *p* < 0.001). ALT was not shorter in patients with positive compared to negative blood cultures (0.63 h vs. 0.94 h; *p* = 0.053). Antimicrobials ordered in the emergency room had a shorter median ALT than in medical wards (0.43 h vs. 1.57 h; *p* < 0.001). After correcting for indication, we found no association between ALT and LoS (*p* = 0.34).

**Conclusions:**

ALT is an easily measurable QI for sepsis. More studies are needed to establish whether ALT is a feasible QI for meningitis and community-acquired pneumonia. For all infections, ALT can be used as process indicator for drug administration.

**Supplementary Information:**

The online version contains supplementary material available at 10.1007/s10096-025-05085-w.

## Introduction

Timely administration of antimicrobial therapy from the moment of presentation at the hospital is associated with decreased in-hospital mortality in patients with sepsis [1–4].

Kashiouris et al. [5] reported that delays of greater than an hour from order to infusion in patients with sepsis were associated with significantly higher mortality rates and that the interval from order to administration was a better predictor of mortality than the interval from triage to first antimicrobial order. Indeed, the point when a clinician prescribes antimicrobials may be a proxy for when patients begin to deteriorate and an association between delay from antimicrobial order to administration and in-hospital mortality has been reported [6]. These data led to the suggestion that antimicrobial order-to-administration time for patients with septic shock may be considered as a potential new quality indicator by the Infectious Diseases Society of America (IDSA) and others [7–9]. Antimicrobial lead time (ALT) is defined as the time from antimicrobial order to administration [5]. ALT is easily measured with the introduction of computerized provider order entry (CPOE) [10] and the objective nature of this measure makes it attractive as process and quality indicator. Quality indicators (QI) are measurable components that give insight in the quality of care and help monitor healthcare processes as well as identify targets for improvement [11]. Process indicators evaluate the efficacy of a system [12]. Deviation from a prespecified range warrants further investigation into the possible cause. Including ALT as a process indicator for antimicrobial stewardship programs in hospitals may facilitate monitoring of the process from antimicrobial order to administration. Few studies have evaluated ALT until now, focusing either on specific infections such as sepsis [5, 6] or only described ALT for the most frequent primary diagnoses per cluster of medical disciplines [13]. In the process of giving appropriate antimicrobial therapy, ALT is an indicator for time elapsed since suspecting or diagnosing an infection and administration. Although ALT has been shown to be a candidate QI for sepsis, data on ALT for different infections, differences between medical specialties and in the emergency room is lacking and the impact of ALT on the length of hospital stay (LoS) is still unknown. Knowledge of such data is necessary before ALT can be introduced as QI. To determine whether ALT would be a possible new process and quality indicator for antimicrobial use, we evaluated the feasibility of retrieving ALT in a tertiary care hospital, the ALT for different infectious diseases and different medical specialties and the association of ALT with LoS.

## Methods

Setting and design.

This retrospective cohort study was conducted in the Erasmus University Medical Center, Rotterdam, the Netherlands, an academic, tertiary care hospital, over a 20-month period. The hospital has 900 beds and 30 clinical care departments. The data were collected as part of the Antibiotic(AB)-assistant clinical trial [14].

### Study population

Adult hospitalized patients receiving systemic antimicrobial therapy for ≥ 24 h during their stay on medical and surgical wards were included after giving informed consent for retrieving demographic, clinical, medication, and microbiological data from the medical charts. Patients receiving antimicrobial prophylaxis, intraoperative antimicrobial therapy, therapy at the outpatient clinic or initiated in another hospital and patients whom passed away were excluded. Additionally, the patient was excluded if the date or time of administration of the antimicrobial were unclear or if the time of administration was deliberately postponed (e.g., communication to nurses that the ordered antimicrobials had to be administered after a medical procedure such as surgery or bronchoalveolar lavage) as it could introduce bias.

### Antimicrobial lead time

Antimicrobial lead time (ALT) was defined as the time in hours from antimicrobial order to antimicrobial administration initiation of the first dose [5]. ALT was automatically calculated by subtracting the drug order date and time from the drug administration date and time. The shortest possible ALT was 0.00 h, including situations when the prescriber made the antimicrobial order after the antimicrobial was administered, which occasionally happens in the emergency room. ALT was calculated for the first therapeutic antimicrobial order of a treatment episode. If more than one antimicrobial was prescribed at the same time, the shortest ALT was used in the analysis as the partially started therapy may already impact clinical outcome such as length of stay.

### From drug order to administration

In Erasmus MC, the electronic health record system (EHR) HiX (Chipsoft version 6.2, 2022, the Netherlands) is used. In the EHR, a prescriber can order a drug, including dosage, dosing frequency, and route of administration. The date and time, and details of the order are registered in the system. Drug orders appear in the EHR dashboard of nurses who distribute and administer the drugs during their medication rounds at fixed time points depending on ward and dosing frequency of the drug. For instance, a drug that is prescribed with a dosing frequency of once daily is administered at 8:00 and a drug that is prescribed with a dosing frequency of three times daily at 8:00, 14:00 and 22:00. During the medication round the nurse scans the barcode on the patient’s bracelet which opens the medical record of the patient in the EHR, scans the barcode of the drug and subsequently administers the drug. Administration generally occurs immediately after the barcode of the drug is scanned. As a result, the date and time of the drug administration is registered. Additionally, nurses can make digital notes for each administration which can give information about the particular drug administration. Administration registration was corrected manually in 14 cases based on these notes.

### Primary outcome

The primary outcome was ALT per infectious disease for which antimicrobial therapy was initiated. If more than one indication was noted and an infectious disease specialist or clinical microbiologist was consulted for this treatment episode within 24 h after prescribing the antimicrobial, we registered the indication that the infectious disease specialist / clinical microbiologist noted. Two or more indications that were equally probable at the time of prescribing were registered as ‘combination indication.’ The registered infectious disease was based on the documented diagnosis by the prescribing physician in the patient’s electronic medical record.

### Secondary outcome

Secondary outcomes were ALT in sepsis vs. other infectious diseases, ALT in patients with positive blood cultures versus no positive blood cultures, difference in ALT between emergency room and ward, recently admitted patients and patients who were already admitted, medical and surgical departments, route of administration and the association of ALT with LoS. LoS was defined as the number of days between antimicrobial order and hospital discharge.

### Data management and statistical analyses

Date and time of hospital admission, department, discharge date and time, antimicrobial order, and antimicrobial administration were automatically aggregated from the EHR. Additionally, drug administration notes were aggregated too. Indication for antimicrobial therapy and if the antimicrobial was prescribed in the emergency room or ward was assessed by manual chart review. All data was anonymized and stored on secure, password-protected hospital servers.

Descriptive and inferential statistics were performed with IBM SPSS Statistics version 28.0.1.0 for Windows (IBM, Armonk, NY, USA). The Mann-Whitney U Test was performed to compare two groups and to compare ALT between several departments the Kruskal-Wallis test was performed followed by the Bonferroni-Dunn post-hoc tests. Linear regression was performed to determine if there was a relation between ALT and LoS. In tests for normalcy, LoS showed no normal distribution and was log-transformed. Two-sided p-values are reported if available.

## Results

In total, 1007 patients were eligible for inclusion. After the exclusion of 7 patients due to unclear administration time of the antimicrobial (6 patients, 0.6%), and deliberate postponement of antimicrobial administration (1 patient, 0.1%), 1000 patients were included (561 men and 439 women). The median age of the included patients was 61 years (interquartile range (IQR) 47–71).

### ALT varies per indication

**Fig. 1:**
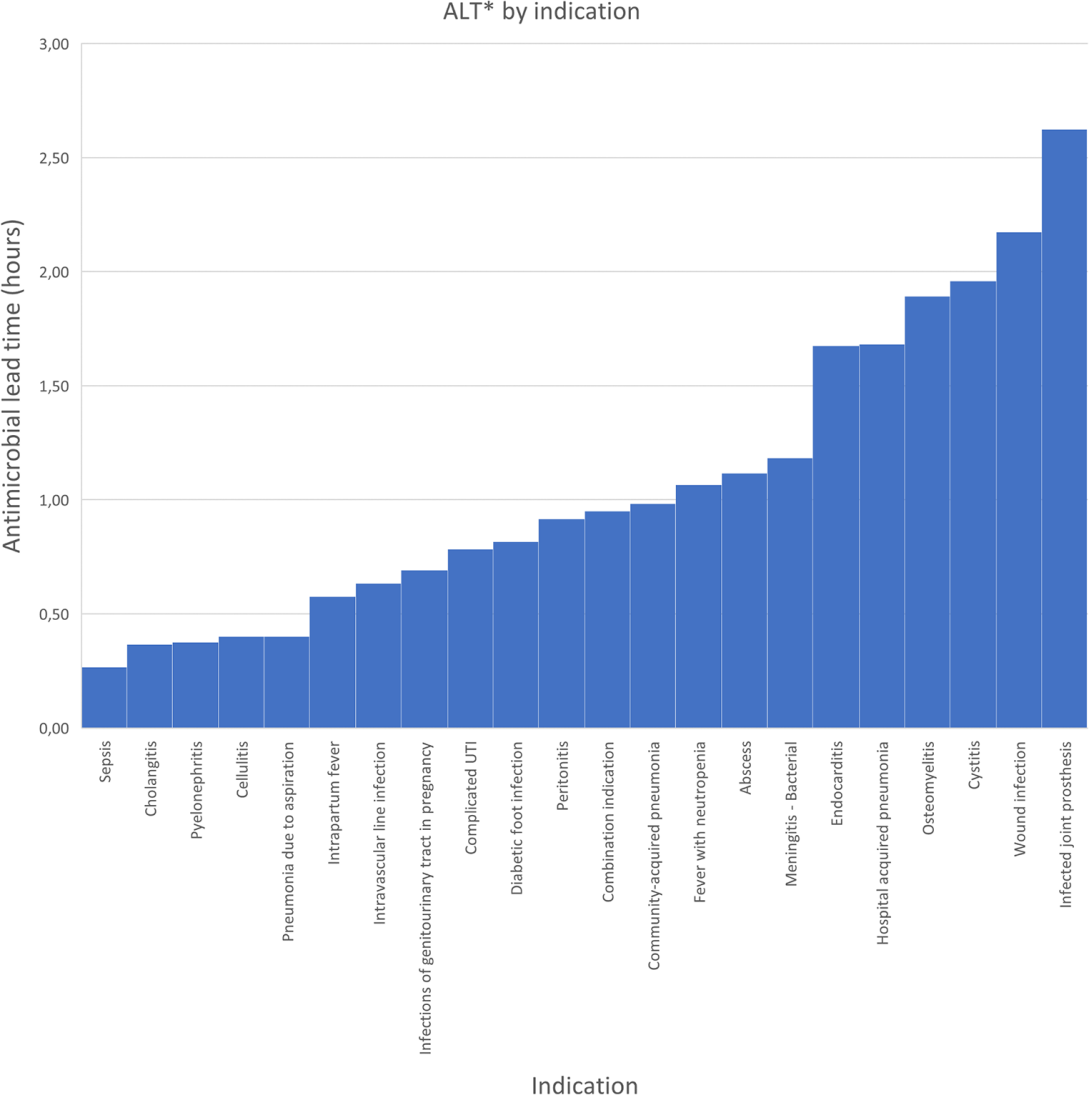
ALT by indication

The median ALT for all patients was 1.05 h (IQR 0.32–3.02). ALT of infectious diseases in ten or more patients are listed in Fig. 1 (Supplementary Table 1). The different indications listed as ‘combination indication’ are listed in Supplementary Table 2. The most frequent indications for antimicrobial use were hospital acquired pneumonia (104/1000) with a median ALT of 1.68 h (IQR 0.73–3.45), fever with neutropenia (74/1000), ALT 1.07 h (IQR 0.64–3.19) and cystitis (72/1000), ALT 1.96 h (IQR 0.56–3.70). Patients with sepsis (65/1000) had the shortest median ALT which was significantly shorter than in the 935/1000 patients with other indications (0.27 h, IQR 0.07–0.67 vs. 1.18 h, IQR 0.37–3.15; *p* < 0.001). The longest median ALT was 2.63 h (IQR 0.88–5.12) for the indication infected joint prosthesis (18/1000 patients).

In 659 patients of whom blood cultures were obtained, the median ALT was shorter than in the 341 patients without blood cultures drawn (median ALT 0.85 h (IQR 0.28–2.42) vs. 1.77 h (IQR 0.43–3.65); *p* < 0.001). In 121 of 659 patients with a positive blood culture (18%), median ALT was not shorter than in the 538 patients with negative blood cultures (82%; median ALT 0.63 h (IQR 0.25–1.88) vs. 0.94 h ((IQR 0.28–2.69); *p* = 0.053).

### ALT varies per medical specialty and time of admission

Antimicrobials ordered at the emergency room (ER) had a significantly shorter median ALT (331/1000 patients; 0.43 h, IQR 0.17–1.43) compared to antimicrobials ordered at medical wards (669/1000 patients; 1.57 h, IQR 0.57–3.43; *p* < 0.001). Patients from 15 different specialties were included; 6 medical departments and 9 surgical departments (Supplementary Table 3). The median ALT was not different for patients at medical and surgical wards (*p* = 0.73). Patients from urology had the shortest median ALT (0.4 h, IQR 0.20–1.37) which differed significantly from the departments orthopaedic surgery (2.32 h, IQR 0.72–4.03; *p* = 0.01) and traumatology (2.28 h, IQR 0.28–1.73; *p* = 0.01). Additionally, differences in median ALT were also found between medical departments such as general medicine with a lower ALT (0.47 h, IQR 0.18–2.12) than pulmonology (1.69 h, IQR 0.56–4.05; *p* = 0.01) and cardiology (1.62 h, IQR 0.7–3.53; *p* = 0.01).

The median ALT for antimicrobials ordered within 24 h of admission (0.77 h (IQR 0.23–2.45; 597 patients) was significantly shorter than for antimicrobials ordered after 24 h of admission (1.67 h (IQR 0.61–3.42; 403 patients) (*p* < 0.001). ALT was also associated with time of the day on which antimicrobials were prescribed; median ALT for antimicrobials ordered for 415 patients during the day 8:00AM < 4:00PM was 1.68 h (IQR 0.46–3.63) and was significantly longer than for antimicrobials ordered for 464 patients in the evening 4:00PM < 12:00PM (0.93 h, IQR 0.28–2.44; *p* < 0.001) and for 121 patients in the night 12:00PM < 8:00AM (0.60 h, IQR 0.22–2.15; *p* < 0.001).

### ALT varies with choice, route and dosing frequency of the antimicrobial

Cefuroxime was the most frequently ordered antimicrobial (289/1000 patients) with a median ALT of 0.60 h (IQR 0.22–2.53). Gentamicin had the shortest median ALT (0.43 h, IQR 0.25–0.93; 32/1000 patients). These drugs are first choice for community acquired sepsis at the Erasmus MC. Conversely, the longest median ALT was found for nitrofurantoin (3.10 h, IQR 1.30–4.92; 32/1000 patients) and ciprofloxacin (2.13 h, IQR 1.00–4.38; 26/1000 patients), both frequently prescribed for urinary tract infections (Supplementary Table 4). Most antimicrobials were prescribed for intravenous administration (876/1000 patients) and had a shorter median ALT than oral antimicrobials (124/1000 patients (0.95 h, IQR 0.28–2.70 vs. 2.41 h, IQR 0.78–4.10; *p* < 0.001)).

Dosing frequencies were once daily (18%), twice daily (13%), three times daily (45%), four times daily (22%) and six times daily (3%). Very long ALTs (i.e. outliers) were noted for antimicrobials with a dosing frequency of once or twice daily; in the 50 patients with the longest ALT (range: 7.87–24.05 h) most patients had a dosing frequency of once daily (42%) or twice daily (16%) compared to three times daily (34%) and four times daily (8%). Of note, the 9 biggest outliers had antimicrobials with a dosing frequency of once a day with an ALT ranging from 17.95 to 24.05 h. For 16 of these 50 patients, more than 1 antimicrobial was ordered simultaneously (Supplementary Table 5). In these patients, antimicrobials with a dosing frequency of once or twice a day had longer ALTs than antimicrobials with a higher dosing frequency. Overall, the median ALT was significantly higher for antimicrobials with a dosing frequency of twice a day (1.82 h, IQR 0.61–4.07) compared to four times per day (1.06 h, IQR 0.38–2.50; *p* = 0.03).

### ALT and length of stay

The median length of stay (LoS) was 7.7 days (IQR 4.5–13.9) for all patients. The shortest median LoS was found in patients with intrapartum fever (2.6 days; median ALT: 0.58 h) and the longest in patients with endocarditis (23.2 days; median ALT: 1.67 h). No association could be demonstrated between ALT and LoS (Supplementary material). For instance, patients with sepsis with a median ALT of 0.27 h had a median LoS of 7.9 days, while patients with infected joint prosthesis with a median ALT of 2.63 h had a median LoS of 7 days. In medical departments median LoS was 8.5 days vs. 6.8 days in surgical departments (*p* < 0.001). In the patient subgroup with positive blood cultures (*n* = 121), we found no association between ALT and LoS (standardized coefficient: 1.06; 95%CI: 0.99–1.13; *p* = 0.10). Additionally, after correcting for the confounder ‘indication’ we found no association between ALT and LoS (standardized coefficient: 1.01; 95%CI: 0.99–1.02; *p* = 0.34).

Despite the exclusion of the patients that died, we did a secondary analysis on these 29 excluded patients and the median ALT was not different from the median ALT of the studied population (2.07 h, IQR 0.53–4.52 vs. 1.05 h, IQR 0.32–3.02; *p* = 0.25).

## Discussion

In this study we explored the potential of ALT as a process and quality indicator for therapeutic antimicrobial prescriptions. We showed that ALT varied by indication, was shorter if blood cultures were obtained and when antimicrobials were administered intravenously. ALT was shorter for patients admitted at the ER than at the ward, but showed no difference between medical and surgical specialties. Furthermore, antimicrobials ordered within 24 h of admission and during the evening and night had a shorter ALT than orders after 24 h of admission and during the day, respectively. ALT was not associated with LoS. This indicates that monitoring ALT is feasible and may help as a process indicator to help identify potential targets for improvement but seems no good candidate as general QI for antimicrobial use for the majority of infectious diseases yet.

So far, there are no guidelines or recommendations on what should be considered as an acceptable ALT. In comparison with the study of Wilder et al., our median ALT for all infections of 1.05 h was high compared to the 48.7% cases with an ALT under 0.5 h for their studied selection of 10 infectious diseases (e.g., COPD with acute exacerbation, pneumonia and sepsis due to *Escherichia coli*) [13]. Delay in antimicrobial therapy in patients with sepsis is associated with increased in-hospital mortality [6, 15]. Guidelines recommend starting antimicrobial therapy in patients with septic shock within 1 h after recognition and within 3 h in septic patients without shock [16]. In the study of Kashiouris et al., the ALT for patients with sepsis was 0–1 h in 13.3%, 1–2 h in 16.1% and Taylor et al. reported a median ALT for patients with sepsis of 0.6 h [6]. In our study, 86.2% of patients with sepsis had an ALT of 0–1 h and 7.7% of 1–2 h with a median ALT of 0.27 h [5]. Furthermore, recommendations have been made for several other infectious diseases regarding the time from disease onset until antimicrobial administration, but these do not include ALT. Immediate antimicrobial therapy is recommended in patients with suspected meningitis [17]. In patients with community-acquired pneumonia, therapy should be started within 4 h of presentation [18]. Awaiting diagnostic results before starting antimicrobial therapy can be feasible for non-life-threatening infectious diseases (as opposed to conditions such as sepsis, meningitis and pneumonia) but it is preferable for hospitalized patients to receive antimicrobial therapy within 3–5 h after onset of symptoms of a suspected infection [19, 20]. However, identifying the exact time of onset of disease is controversial, which impedes determining time from infection presentation to antimicrobial administration [21, 22]. ALT uncovers the objective part of this metric as it is an concrete interval that can easily be measured. Based on our study, and these previously published studies, ALT might be a good potential QI for patients with sepsis, meningitis and community-acquired pneumonia, although the variation associated with admission ward, antimicrobial and prescription time should be taken into account. For most other infectious diseases there are no guidelines regarding the timing of antimicrobial therapy or studies on the association of timing of antimicrobial therapy and clinical outcomes such as mortality and length of stay. For these infectious diseases, ALT can be utilized as a process indicator to monitor the process from antimicrobial order to administration which enables identification of potential barriers of antimicrobial administration. A longer than expected median ALT for infectious diseases on a certain ward could trigger further investigation into systematic barriers and improve operational efficiency.

An additional finding was that dosing frequency influenced ALT. Once a day dosing had the longest ALT in our study. This may be due to how EHR systems are designed. In our hospital, standard administration times are included in the EHR (HiX (Chipsoft)). For the majority of drugs, including antimicrobials, a dosing frequency of once daily results in an administration time of 8.00 AM, which generally results in a first planned administration the following day. The prescribing physician may change the time of the first administration by clicking on a clock icon to advance the administration but may forget to use it or even does not know this function which can result in very long ALT. This underlines the importance of introductory EHR courses for prescribers. Alternatively, these outliers may indicate the necessity for improvements of the EHR that prevent excessive ALT due to suboptimal system design. Nurse workflow adjustments could also ensure ALT stays within an acceptable range for example by increasing the number of administration times for once-daily dosed antimicrobials.

ALT was shorter if blood cultures were obtained, antimicrobials were administered intravenously or on the ER which may indicate more severe disease. Additionally, the ER has specific operational efficiencies compared to the ward such as no delays due to designated medication rounds when the ordered antimicrobial is administered. During after hours, clinicians are generally engaged in delivering urgent care. Consequently, antimicrobials ordered at night will be administered in a timely fashion and not await the next medication round. Moreover, availability of a drug, preparation time and experience of the nurse could impact ALT. Similarly, certain departments may encounter infections more frequently in their inpatient population possibly resulting in different availability and experience with antimicrobials and could explain the differences in ALT between departments. Therefore, utilizing ALT as a universal process or quality indicator for specific infections would be more sensible. Departments could learn from each other on how to improve operational efficiency and thereby potentially reduce ALT.

### Strengths and limitations

This is the first study that evaluated ALT per indication as a potential QI and process indicator for all infectious diseases. Limitations are that we assumed that disease severity of a certain indication and time to presentation were equal for all infections. By only using an indication noted in the EHR within 24 h after prescription of antimicrobial therapy, we think our data gives a good indication about the value and limitations of ALT as potential QI. The retrospective nature of our study and reliance on accurate EHR documentation could lead to systemic biases. The single center study design and differences in data recording systems and operational workflows across centers may affect the generalizability and reproducibility of our findings. However, in an ever more digital and automated world, ALT will probably be more easily and widespread adapted in hospitals with EHRs. Additionally, dosing and administration errors may occur which could lead to a shorter ALT, but would commonly be noted in the administration registration. Although we included 1000 patients, for the majority of indications less than 50 patients were included which may have impeded detection of association between ALT and LoS for certain infections. Furthermore, it is possible that patients whom received oral antimicrobials did not swallow the pill immediately when it was offered. Therefore, ALT for oral antimicrobials may have been longer than we calculated. However, only prompt administration of intravenous antimicrobials for severe infectious diseases such as sepsis and meningitis has been recommended in guidelines [16, 17] and no such data exist for oral antimicrobials.

## Conclusions

ALT is confirmed to be an easy to measure QI for sepsis, although we could not show an impact on outcome. In addition, more studies are needed to establish whether ALT is a feasible QI for meningitis and community-acquired pneumonia, that have guidelines on time from disease onset until antimicrobial administration. For all infections, our data suggests ALT seems to be a good process indicator for drug administration.

## Electronic supplementary material

Below is the link to the electronic supplementary material.

Supplementary Material 1

## Data Availability

Data is provided within the manuscript or supplementary information files.
