# Supplementary material for: The feasibility of antimicrobial lead time as process and quality indicator for hospitals
Source: Eur J Clin Microbiol Infect Dis. 2025 Mar 7;44(5):1177–83. doi: 10.1007/s10096-025-05085-w (PMC12062096; doi:10.1007/s10096-025-05085-w)
Supplement: Supplementary file 1 — Supplementary Material 1 [file 10096_2025_5085_MOESM1_ESM.docx]

**Supplementary files**

Supplementary Table 1. ALT by indication

| **Indication** | **Patients** | **ALT^a^ (IQR) (hours)** | **LoS^a^ d (IQR) (days)** |
| --- | --- | --- | --- |
| Abscess | 26 | 1.12 (0.32 - 2.42) | 8 (5.5 - 10.2) |
| Cellulitis | 22 | 0.4 (0.17 - 1.26) | 5.9 (3.9 - 9.5) |
| Cholangitis | 51 | 0.37 (0.15 - 1.38) | 4.4 (3.3 - 6.9) |
| Combination indication | 19 | 0.95 (0.36 - 3.3) | 9.2 (6.8 - 24.9) |
| Community-acquired pneumonia | 61 | 0.98 (0.25 - 3.6) | 9.7 (6.1 - 14.5) |
| Complicated UTI | 49 | 0.78 (0.23 - 2.27) | 6.7 (4.3 - 9.8) |
| Cystitis | 72 | 1.96 (0.56 - 3.7) | 7.4 (4.1 - 12.8) |
| Diabetic foot infection | 11 | 0.82 (0.6 - 1.82) | 9.9 (6 - 15.3) |
| Endocarditis | 10 | 1.67 (0.83 - 2.32) | 23.2 (12.5 - 30.8) |
| Intrapartum fever | 10 | 0.58 (0.25 - 1.83) | 2.6 (2.4 - 2.9) |
| Hospital acquired pneumonia | 104 | 1.68 (0.73 - 3.45) | 8.1 (5.1 - 12.7) |
| Infected joint prosthesis | 18 | 2.63 (0.88 - 5.12) | 7 (5.2 - 9.7) |
| Infections of genitourinary tract in pregnancy | 26 | 0.69 (0.23 - 1.76) | 4 (2.4 - 4.9) |
| Intravascular line infection | 13 | 0.63 (0.5 - 2.37) | 11.8 (8.1 - 16) |
| Meningitis – Bacterial | 30 | 1.18 (0.46 - 2.85) | 12.3 (7.6 - 21.4) |
| Fever with neutropenia | 74 | 1.07 (0.64 - 3.19) | 13.5 (7.1 - 24) |
| Osteomyelitis | 30 | 1.89 (0.62 - 3.94) | 7.8 (5 - 16.4) |
| Peritonitis | 29 | 0.92 (0.43 - 1.88) | 11.9 (6.6 - 15) |
| Pneumonia due to aspiration | 31 | 0.4 (0.24 - 2.09) | 10 (5.3 - 17.9) |
| Pyelonephritis | 28 | 0.38 (0.09 - 1.04) | 5.3 (3.5 - 6.6) |
| Sepsis | 65 | 0.27 (0.07 - 0.67) | 7.9 (4.8 - 11.3) |
| Wound infection | 46 | 2.17 (0.33 - 3.86) | 7.1 (4.4 - 11.1) |

Abbreviations: ALT, antimicrobial lead time; IQR, interquartile range; LoS, length of stay.
^a^Median.

Supplementary Table 2. List of combination indications

| Indication 1 | Indication 2 | Count |
| --- | --- | --- |
| Arthritis | Endocarditis | 1 |
| Endocarditis | Spondylodiscitis | 1 |
| Hospital acquired pneumonia | Complicated UTI | 2 |
| Hospital acquired pneumonia | Cholangitis | 1 |
| Hospital acquired pneumonia | Cystitis | 2 |
| Infected haematoma | Cystitis | 1 |
| Mediastinitis | Mediastinitis | 1 |
| Meningitis - Bacterial | Cystitis | 1 |
| Mycotic aneurysm | Osteomyelitis | 1 |
| Community-acquired pneumonia - organism unspecified | Peritonitis | 1 |
| Community-acquired pneumonia - organism unspecified | Pyelonephritis | 1 |
| Community-acquired pneumonia - organism unspecified | Cystitis | 3 |
| Community-acquired pneumonia - organism unspecified | Cholangitis | 1 |
| Pneumonia due to aspiration | Cellulitis | 1 |
| Pyothorax | Complicated UTI | 1 |

Supplementary Table 3: ALT in specialty and department: a stepwise comparison

| Specialty | Patients | ALT^a^ (IQR) (hours) | P^b^ | LoS^a^ (IQR) (days) |
| --- | --- | --- | --- | --- |
| Medical (6 departments) | 552 | 1.03 (0.33 - 2.77) |  | 8.5 (4.9 - 15.9) |
| Surgical (9 departments) | 448 | 1.12 (0.32 - 3.1) | 0.70 | 6.8 (4 - 10.8) |
| Gastroenterology | 95 | 0.57 (0.18 - 1.72) |  | 5 (3.6 - 11.2) |
| Pulmonology | 96 | 1.69 (0.56 - 4.05) | 0.03 | 8.3 (5 - 13.7) |
| Cardiology | 77 | 1.62 (0.7 - 3.53) | 0.04 | 10.8 (6.1 - 19.5) |
| Cardiothoracic surgery | 61 | 1.83 (0.82 - 3.63) | 0.02 | 8.1 (4.9 - 11.5) |
| Gynaecology | 76 | 0.57 (0.13 - 1.54) |  | 4.1 (2.7 - 6.4) |
| Pulmonology | 96 | 1.69 (0.56 - 4.05) | <0.01 | 8.3 (5 - 13.7) |
| Cardiology | 77 | 1.62 (0.7 - 3.53) | <0.01 | 10.8 (6.1 - 19.5) |
| Traumatology | 53 | 2.28 (0.63 - 4.03) | <0.01 | 6.8 (3.8 - 13) |
| Cardiothoracic surgery | 61 | 1.83 (0.82 - 3.63) | <0.01 | 8.1 (4.9 - 11.5) |
| Orthopaedic surgery | 36 | 2.32 (0.72 - 4.03) | 0.01 | 7.4 (5.9 - 9.2) |
| General medicine | 117 | 0.57 (0.18 - 2.12) |  | 7.2 (4.8 - 12.7) |
| Pulmonology | 96 | 1.69 (0.56 - 4.05) | 0.01 | 8.3 (5 - 13.7) |
| Cardiology | 77 | 1.62 (0.7 - 3.53) | 0.01 | 10.8 (6.1 - 19.5) |
| Traumatology | 53 | 2.28 (0.63 - 4.03) | 0.02 | 6.8 (3.8 - 13) |
| Cardiothoracic surgery | 61 | 1.83 (0.82 - 3.63) | <0.01 | 8.1 (4.9 - 11.5) |
| Orthopaedic surgery | 36 | 2.32 (0.72 - 4.03) | 0.04 | 7.4 (5.9 - 9.2) |
| Urology | 80 | 0.4 (0.2 - 1.37) |  | 6 (3.6 - 8.1) |
| Pulmonology | 96 | 1.69 (0.56 - 4.05) | <0.01 | 8.3 (5 - 13.7) |
| Cardiology | 77 | 1.62 (0.7 - 3.53) | <0.01 | 10.8 (6.1 - 19.5) |
| Traumatology | 53 | 2.28 (0.63 - 4.03) | 0.01 | 6.8 (3.8 - 13) |
| Cardiothoracic surgery | 61 | 1.83 (0.82 - 3.63) | 0.01 | 8.1 (4.9 - 11.5) |
| Orthopaedic surgery | 36 | 2.32 (0.72 - 4.03) | 0.01 | 7.4 (5.9 - 9.2) |

Abbreviations: ALT, antimicrobial lead time; IQR, interquartile range; LoS, length of stay.

**^a^**Median ALT

**^b^**Significant values adjusted by the Bonferroni-Dunn post-hoc tests

Supplementary Table 4. ALT per most frequently ordered antimicrobial

| **Antimicrobial** | **Number of administrations** | **ALT^a^ (IQR) (hours)** |
| --- | --- | --- |
| Cefuroxime | 289 | 0.6 (0.22 - 2.53) |
| Meropenem | 160 | 1.06 (0.33 - 3.55) |
| Piperacillin/tazobactam | 146 | 1.31 (0.58 - 3.01) |
| Amoxicillin/clavulanic acid | 128 | 0.85 (0.25 - 2.28) |
| Flucloxacillin | 49 | 1.6 (0.47 - 3.17) |
| Ceftriaxone | 40 | 0.94 (0.27 - 2.92) |
| Nitrofurantoin | 32 | 3.1 (1.3 - 4.92) |
| Gentamicin | 32 | 0.43 (0.25 - 0.93) |
| Ciprofloxacin | 26 | 2.13 (1 - 4.38) |
| Vancomycin | 21 | 1.58 (0.67 - 2.67) |
| Amoxicillin | 18 | 1.18 (0.62 - 2.98) |
| Metronidazole | 16 | 1.17 (0.42 - 2.49) |
| Levofloxacin | 10 | 1.89 (0.65 - 3.27) |

Abbreviations: ALT, antimicrobial lead time.
^a^Median.

Supplementary Table 5. Dosing frequency for longest ALT with two drugs ordered at the same time

| **Antimicrobial 1** | **Dosing frequency** | **ALT antimicrobial 1 (hours)** | **Antimicrobial 2** | **Dosing frequency** | **ALT antimicrobial 2 (hours)** | **Difference in ALT antimicrobial 1 & 2 (hours)** |
| --- | --- | --- | --- | --- | --- | --- |
| Ceftriaxone | 1 | 18.50 | Metronidazole | 3 | 1.90 | 16.60 |
| Ceftriaxone | 1 | 18.43 | Ciprofloxacin | 2 | 7.78 | 10.65 |
| Cefuroxime | 3 | 15.92 | Metronidazole | 3 | 5.65 | 10.27 |
| Cefuroxime | 3 | 13.18 | Metronidazole | 3 | 13.18 | 0.00 |
| Ceftriaxone | 1 | 12.77 | Doxycycline | 2 | 1.75 | 11.02 |
| Ceftriaxone | 1 | 11.83 | Ciprofloxacin | 2 | 0.38 | 11.45 |
| Ciprofloxacin | 2 | 11.00 | Clindamycin | 3 | 1.45 | 9.55 |
| Ceftriaxone | 1 | 10.78 | Ciprofloxacin | 3 | 1.78 | 9.00 |
| Ceftriaxone | 1 | 10.13 | Ciprofloxacin | 2 | 1.30 | 8.83 |
| Piperacillin/tazobactam | 4 | 9.82 | Gentamicin | 1 | 0.48 | 9.33 |
| Ceftriaxone | 1 | 9.38 | Metronidazole | 3 | 1.32 | 8.07 |
| Levofloxacin | 2 | 9.25 | Metronidazole | 3 | 0.43 | 8.82 |
| Metronidazole | 3 | 8.82 | Cefuroxime | 2 | 8.78 | 0.03 |
| Colistin | 3 | 8.60 | Piperacillin/tazobactam | 4 | 4.68 | 3.92 |
| Cefuroxime | 3 | 8.45 | Metronidazole | 3 | 8.45 | 0.00 |
| Cefuroxime | 2 | 7.88 | Gentamicin | 1 | 0.30 | 7.58 |

Abbreviations: ALT, antimicrobial lead time.
